# Assessing Fecal Microbial Diversity and Hormone Levels as Indicators of Gastrointestinal Health in Reintroduced Przewalski’s Horses (*Equus ferus przewalskii*)

**DOI:** 10.3390/ani14172616

**Published:** 2024-09-09

**Authors:** Zhenghao Li, Zhengwei Luo, Defu Hu

**Affiliations:** School of Ecology and Nature Conservation, Beijing Forestry University, Qinghua East Road 35, Beijing 100083, China; a2358905142@163.com (Z.L.); luozw114688@gmail.com (Z.L.)

**Keywords:** Przewalski’s horse, diarrhea, fecal microbial, 16S rDNA, cortisol, triiodothyronine (T3), immunoglobulin A

## Abstract

**Simple Summary:**

Upon release into the wild, Przewalski’s horses (*Equus ferus przewalskii*) undergo adaptation to diverse habitats and dietary patterns. These environmental shifts prompt adaptive alterations in fecal microbiota, yet render them vulnerable to gastrointestinal ailments, notably diarrhea. However, the interplay between fecal microbiota, hormones, and diarrhea in Przewalski’s horses remains largely unexplored. An analysis of fecal samples revealed substantial shifts in the composition and diversity of fecal microbiota, as well as fluctuations in hormone levels during episodes of diarrhea in Przewalski’s horses. These findings highlight the potential significance of fecal microbiota and hormonal dynamics as factors contributing to diarrhea occurrences in Przewalski’s horse populations.

**Abstract:**

Diarrhea serves as a vital health indicator for assessing wildlife populations post-reintroduction. Upon release into the wild, wild animals undergo adaptation to diverse habitats and dietary patterns. While such changes prompt adaptive responses in the fecal microbiota, they also render these animals susceptible to gastrointestinal diseases, particularly diarrhea. This study investigates variations in fecal microorganisms and hormone levels between diarrhea-afflicted and healthy Przewalski’s horses. The results demonstrate a significant reduction in the alpha diversity of the fecal bacterial community among diarrheal Przewalski’s horses, accompanied by notable alterations in taxonomic composition. Firmicutes, Proteobacteria, and Bacteroidetes emerge as dominant phyla across all fecal samples, irrespective of health status. However, discernible differences in fecal bacterial abundance are observed between healthy and diarrhea-stricken individuals at the genus level, specifically, a diminished relative abundance of Pseudobutyrivibrio is observed. The majority of the bacteria that facilitate the synthesis of short-chain fatty acids, *Christensenellaceae_R_7_group* (*Christensenellaceae*), *NK4A214_group* (*Ruminococcus*), *Lachnospiraceae_XPB1014_group* (*Lachnospiraceae*), [*Eubacterium*]_*coprostanoligenes_group* (*Eubacterium*), *Rikenellaceae_RC9_gut_group*, *Lachnospiraceae_AC2044_group* (*Lachnospiraceae*), and *Prevotellaceae_UcG*_001 (*Prevotella*) are noted in diarrhea-affected Przewalski’s horses, while Erysipelotrichaceae, Phoenicibacter, Candidatus_Saccharimonas (*Salmonella*), and Mogibacterium are present in significantly increased amounts. Moreover, levels of immunoglobulin IgA and cortisol are significantly elevated in the diarrhea group compared with the non-diarrhea group. Overall, this study underscores substantial shifts in fecal bacterial diversity, abundance, and hormone levels in Przewalski’s horses during episodes of diarrhea.

## 1. Introduction

Diarrhea represents a significant contributing factor to the decline and mortality of wildlife populations [1]. Previous studies have consistently demonstrated the prevalence of diarrhea in a range of wildlife species. Given the deleterious effects of diarrhea on wildlife, it is of paramount importance to investigate the causes and treatment of this condition. A multitude of studies have highlighted the pivotal role of the microbiome in the prevention, management, and diagnosis of diarrhea [2,3]. Dysregulation of gut flora can precipitate a spectrum of gastrointestinal disorders, encompassing diarrhea, enteritis, and irritable bowel syndrome [4,5]. Recent investigations have demonstrated that episodes of diarrhea in goats and giraffes are associated with significant alterations in the composition of the fecal microbiota, which correlate with elevated mortality rates [6]. Likewise, research on the fecal microbiota of yaks and mice has indicated that dysbiosis of gut microbes could be a contributing factor to diarrhea [7,8]. Concurrently, the interplay between microbes and hormones exerts a significant influence on the metabolism, immunity, and behavior of the host organism. This interaction is bidirectional, with the microbiome exerting an influence on and being influenced by host hormones. The microbiome exerts a significant influence on the regulation of hormones, which span across various functional categories and exert a broad spectrum of effects on host behavior, metabolism, appetite, growth, reproduction, and immunity [9].

The microbiome’s impact on the host’s hormone levels can be both direct and indirect. Direct influence occurs when the microbiome produces hormones, while indirect influence encompasses the modulation of adrenocortical function, regulation of stress responses, and modulation of inflammatory and immune responses [10,11]. Triiodothyronine (T3) plays a crucial role in nutritional physiology, governing the basal metabolic rate of the body and serving as a reliable indicator for assessing the nutritional status of animals. Hence, cortisol, triiodothyronine T3, and immunoglobulin IgA were chosen as hormone markers for detection.

*Equus ferus przewalskii* was previously believed to be extinct in China and Mongolia [12]. In 1945, a mere 31 horses survived in captivity, but concerted conservation endeavors across multiple nations saw their numbers burgeon to over 1500. China undertook its inaugural reintroduction of the Przewalski’s horse in 2001, successfully releasing 206 horses into the Kalamaili Mountain Hoofed Animal Nature Reserve in Xinjiang province. By 2023, the population of Przewalski’s horses in the reserve had swelled to 386 individuals [13,14]. Przewalski’s horses undergo a crucial period of adaptation to varied habitats and a diverse array of food sources upon release into the wild. This shift in living conditions can trigger an adaptive response in their fecal flora while also rendering them vulnerable to a range of gastrointestinal diseases, notably diarrhea [15,16,17,18]. Nevertheless, the potential interplay between the fecal microbiota, hormones, and diarrhea in Przewalski’s horses remains to be elucidated. The objective of this study was to comprehensively analyze and compare the composition and structure of the fecal microbiota, along with fecal hormone levels, between healthy and diarrheal Przewalski’s horses. The objective of this study is to provide a scientific foundation for the prevention and management of diarrhea in Przewalski’s horses. This will enhance the overall health of the reintroduced population. To achieve this, we employed high-throughput 16S rRNA gene sequencing technology and the enzyme-linked immunosorbent assay.

## 2. Materials and Methods

### 2.1. Animals and Sample Collection

In total, 14 Przewalski’s horses (7 healthy and 7 experiencing diarrhea) were procured from the hoofed nature reserve of Karamaili Mountain in Xinjiang, China, for the experiment. These horses were reintroduced into the reserve in 2020 after being reared using standardized breeding practices, with immunization being observed at the Xinjiang Przewalski’s Horse Conservation Center. Additionally, a qualified veterinarian conducted an assessment to confirm the health status of each horse before procuring stool samples. Fresh stool specimens weighing approximately 200 g were promptly collected post-defecation, encompassing both healthy and diarrheal samples, and immediately placed in sterile plastic containers for preservation in liquid nitrogen during transportation to the laboratory, where they were stored at −80 °C for subsequent analysis.

### 2.2. Gene Amplicon Sequencing of 16 SrDNA

Total genomic DNA was extracted from 14 fecal Przewalski’s horse samples using TGuide S96 Magnetic Soil/Stool DNA Kit (Tiangen Biotech (Beijing) Co., Ltd., Beijing, China) in accordance with the manufacturer’s instructions. Hypervariable regions V3-V4 of the bacterial 16S rRNA gene were amplified with primer pair 338F (5′-ACTCCTACGGGAGGCAGCA-3′) and 806R (5′-GGACTACHVGGGTWTCTAAT-3′). PCR products were checked on agarose gel and purified through the Omega DNA purification kit (Omega Inc., Norcross, GA, USA). The purified PCR products were collected, and paired ends (2 × 250 bp) were performed on the Illumina Novaseq 6000 platform (Source from Illumina, San Diego, CA, USA).

### 2.3. Bioinformatics and Data Analysis

The initial data from Illumina MiSeq sequencing were evaluated for quality to obtain valid data. In brief, Trimmomatic (v0.33) and Cutadapt (1.9.1) were used to screen the original data containing problem sequences (including short, unqualified, and mismatched sequences) and remove the primer sequences to obtain clean reads. Usearch (vlO) was used to splice clean reads, and then the spliced sequences were screened twice based on the sequence length range. Then, UCHIME (v4.2) was used to identify and eliminate chimeric sequences to obtain the final valid reads. The valid reads that passed the quality check were clustered, and OTUs were divided based on 97% similarity. In addition, a Venn diagram was generated to characterize the distribution and abundance of bacterial OTUs in each sample. To further investigate the changes in intestinal microbial diversity and abundance during diarrhea, multiple alpha diversity indices were calculated based on OTU distribution. Principal component analysis was also performed to dissect the β diversity of intestinal bacteria between the two groups. The sequencing depth and evenness of each sample were evaluated via rank abundance and the sparse curve. Metastats and LEfSe analysis were used to identify the differential bacterial taxa associated with diarrhea exposure. SPSS statistical program (v20.0) was used for data analysis, and *p* values (mean ± SD) < 0.05 were considered statistically significant.

### 2.4. Hormone Extraction and Detection

Extraction was conducted following the method outlined by Wasser with some modifications. Briefly, 1 g of fresh feces was weighed and placed into a 15 mL centrifuge tube. Subsequently, 10 mL of 90% ethanol aqueous solution was added, and the mixture was vigorously vortexed for 5 min, followed by being incubated at 60 °C for 20 min. After centrifugation at 2500 rpm for 20 min, the supernatant was carefully collected. To the sediment, 5 mL of ethanol solution of the same concentration was added. After shaking for 2 min and another round of centrifugation, the two supernatants were combined. The combined supernatants were then evaporated at 60 °C, reconstituted with 1 mL of PBS, and stored at −20 °C for further analysis.For moisture content determination, 1 g of wet feces was weighed and dried in a container.

The immunoglobulin extraction method was optimized based on the methodology established by Peters et al. [19]. Briefly, 1 g of freshly ground wet feces was homogenized in a 15 mL plastic centrifugal tube containing 10 mL of PBS buffer. The resulting mixture underwent vortexing for 10 min at low speed and subsequent centrifugation at room temperature for 20 min at 2000 r/min. Following this, 2 mL of supernatant was carefully transferred to a new tube and subjected to ultra-low-temperature centrifugation (10,000 r/min) using an ordinary high-speed centrifuge for an additional 20 min. Subsequently, 1.5 mL of supernatant was aliquoted into a new tube and stored below −20 °C for further analysis.

Commercial enzyme-linked immunosorbent assay (ELISA) kits from Enzo Life Sciences and Arbor Assays were utilized for quantifying cortisol metabolites (Cortisol Kit #ADI-900-071; https://www.enzolifesciences.com) (accessed on 16 April 2024) and thyroid metabolites (T3 Kit #K056-H1; https://www.arborassays.com) (accessed on 16 April 2024), respectively. Immunoglobulin metabolites were extracted utilizing the immunoglobulin kit manufactured by North Biotechnology (http://www.bnibt.com/) (accessed on 16 April 2024). These antibodies were chosen based on their successful application in fecal hormone metabolite quantification across various mammalian species [20].

### 2.5. Verification of Determination

The validation of the analysis entails standard measurements of parallelism and accuracy. Parallelism is evaluated through serial dilution of the combined samples in the assay buffer provided in each kit. For both cortisol and T3, pure solutions (undiluted) and seven dilutions (ranging from 1:2 to 1:64) are included. This test determines the sample dilution at 50% binding, representing the most accurate part of the hormone curve measured. Accuracy is assessed according to each hormone standard in the mixed sample at 50% binding. All combined validation, standard, and control samples were tested in two replicates for parallelism and in three replicates for accuracy. Acceptance criteria included a batch-to-batch coefficient of variation (CV) of <15% and an intra-batch CV of <10%. Each plate includes complete standard curves, non-specific binding wells, total reactive wells, and blank wells.

## 3. Results

### 3.1. Sequences Analyses

In this study, high-throughput sequencing analysis was conducted on seven healthy and seven diarrheic fecal samples. Following optimization of the raw data, 1,120,777 high-quality sequences in total were obtained from the 14 samples (see Table 1). Additionally, the number of valid sequences in healthy Przewalski’s horses ranged from 799.70 to 802.56 (healthy fecal samples: CY1–7), whereas in the diarrhea group, it ranged from 797.87 to 801.35 (diarrheic fecal samples: DY1–7).

**Figure 1:**
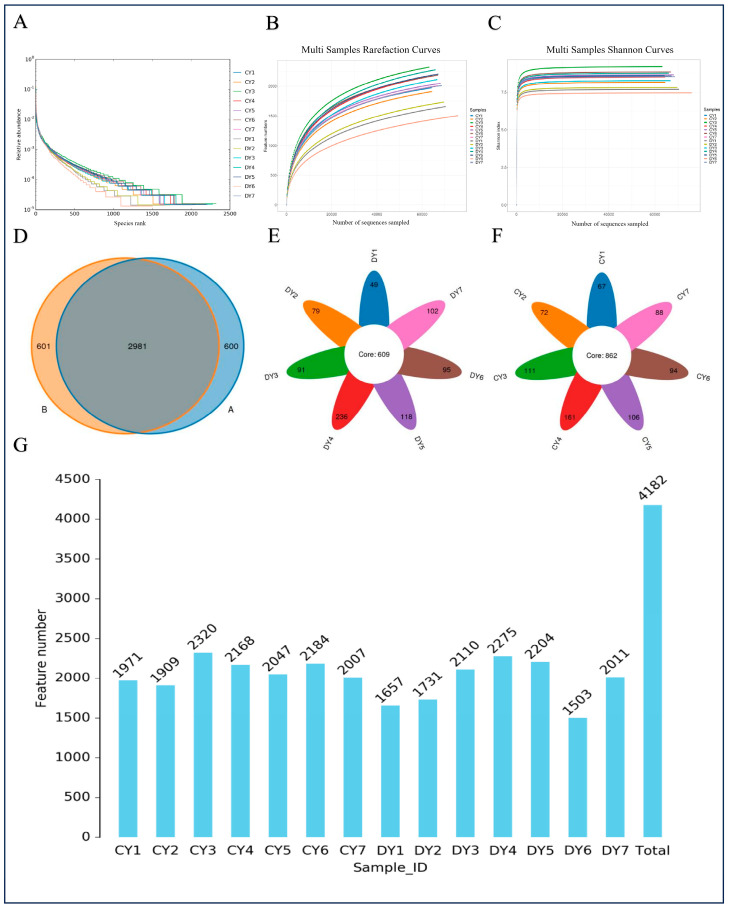
Feasibility analysis of sequencing data (healthy: CY; diarrheic: DY). The sequencing depth and evenness of fecal flora can be assessed using sparsity (**A**,**B**) and grade abundance curves (**C**). The distribution of OTUs in intestinal bacteria from different samples is represented by (**D**–**F**). Additionally, the number of OTUs in the sample can also be determined (**G**).

The Chao1, Shannon, and rank abundance curves exhibited a saturation trend, indicating sufficient depth and evenness of sequencing (see Figure 1A–C). High-quality sequences, characterized by 97% nucleotide sequence similarity, were clustered into operational taxonomic units (OTUs). In total, 4182 OTUs were identified in the gut bacterial community, ranging from 1657 to 2320 in each sample (see Figure 1E). Furthermore, 3582 and 3581 OTUs were observed in healthy and diarrheic Przewalski’s horses, respectively, with a total of 2981 OTUs shared between the two groups, representing approximately 71.28% of the total OTUs (see Figure 1D).

### 3.2. Analysis of Microbial Diversity in the Healthy and Diarrheic Horses

**Figure 2:**
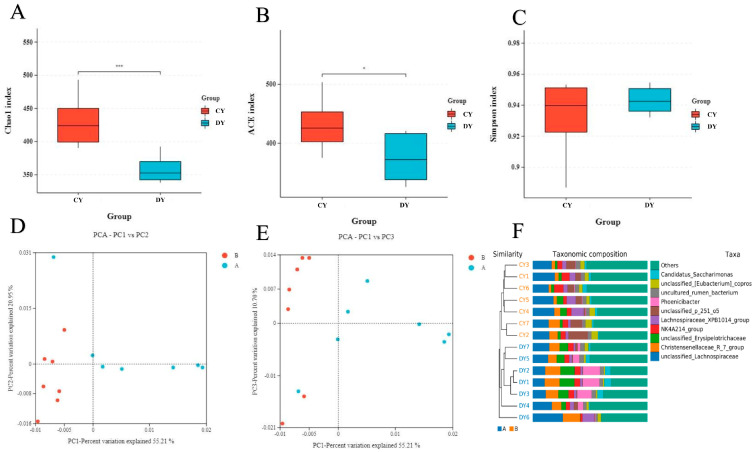
Comparative analysis of intestinal flora α and β diversity between healthy (group: CY) and diarrheic (group: DY) Przewalski’s horses. (**A**–**C**) Chao, ACE, and Shannon indices, respectively. (**D**,**F**) PCoA plots based on (**E**) weighted UniFrac distance and (**F**) unweighted UniFrac distance. (**F**) Cluster analysis plot (* *p* ≤ 0.05,*** *p* ≤ 0.001).

Chao1, ACE, Shannon, and Good’s coverage indices were calculated to assess the alpha diversity of the microbial community. Good coverage estimates ranged from 99.88% to 99.96% for all samples, indicating satisfactory coverage. The average Chao1 and ACE indices in healthy Przewalski’s horses were 2397.18 and 2287.90, respectively, while in the diarrhea group, they were 2287.90 and 2302.63 (see Figure 2A,B). Additionally, the average Shannon indices in healthy and diarrheic Przewalski’s horses were 8.65 and 8.16, respectively (see Figure 2C). Statistical analysis revealed that the Chao1 and ACE diversity indices in healthy Przewalski’s horses were significantly higher than those in the diarrhea group. These results suggest notable differences in the abundance and diversity of intestinal microbial populations between healthy and diarrheic Przewalski’s horses.

Furthermore, principal coordinates analysis (PCoA) scatter plots of fecal flora exhibited distinct separation between samples from healthy and diarrheic Przewalski’s horses. This separation was consistent with the results of the unweighted pair group method with arithmetic mean (UPGMA), indicating significant alterations in the major composition of the intestinal microbial population (see Figure 2D–F).

### 3.3. Composition Analysis of the Gut Microbial Community in Healthy and Diarrheic Horses

**Figure 3:**
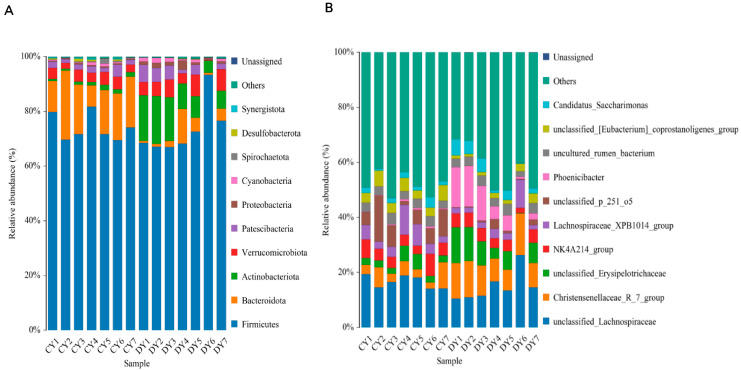
Proportions of (**A**) phylum and (**B**) genus of predominant bacteria in healthy and diarrheic horses (healthy: CY; diarrheic: DY).

The fecal microbial community composition of healthy and diarrheic Przewalski’s horses was assessed at various taxonomic levels. At the phylum level, Firmicutes (73.98% and 73.67%), Bacteroidetes (16.31% and 3.71%), Actinobacteria (1.26% and 11.10%), and Verrucomicrobia (3.71% and 5.12%) emerged as the dominant phyla in both healthy and diarrheic *P. przewalskii* horses, irrespective of health status (see Figure 3A).

Furthermore, comparisons were made between fecal microbiota at the phylum and genus levels in healthy and diarrheic *P. przewalskii* horses. At the phylum level, the relative abundance of Actinobacteria and Bacteroidetes in healthy *P. przewalskii* horses was significantly higher than that in diarrheic *P. przewalskii* horses, while that of Acinetobacter, Verrucomicrobia, Flavobacteria, Spirochaetes, Firmicutes, Patescibacteria, and Proteobacteria was lower. Additionally, other phyla such as Proteobacteria (0.41% and 1.4%), Cyanobacteria (0.54% and 1.02%), Spirochaetes (0.49% and 0.72%), and Desulfobacterota (0.03% and 0.81%) were lower in abundance in both groups.

**Figure 4:**
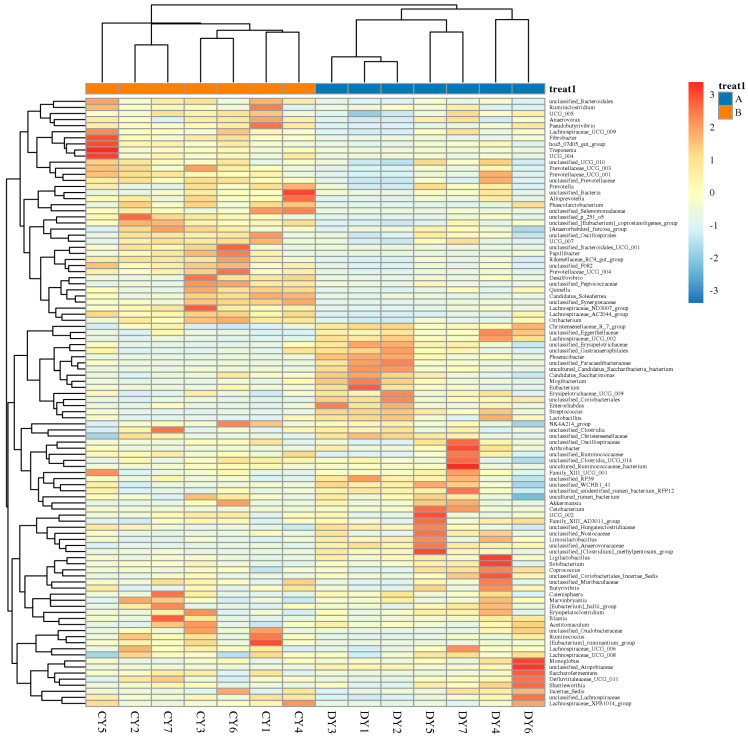
Heatmaps of genus–level clustering of microbial communities in healthy (Group A) and diarrhea (Group B) Przewalski’s horses.

**Figure 5:**
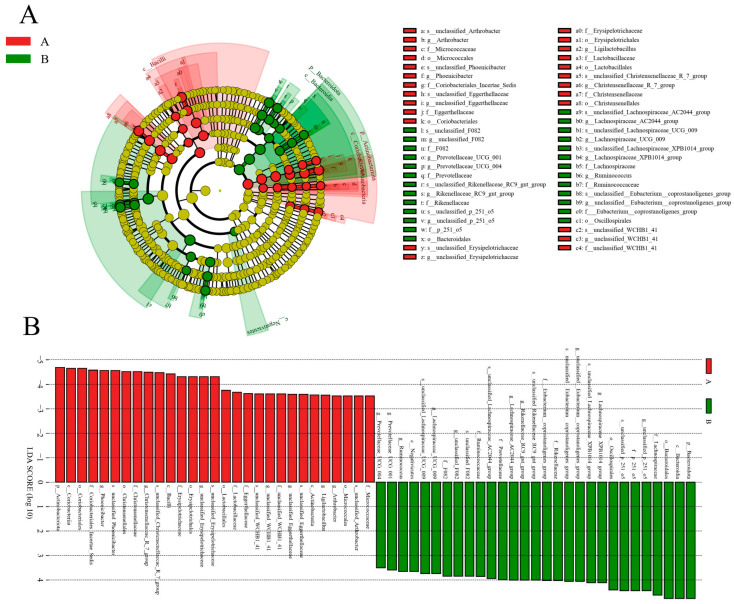
Differential biomarkers of diarrhea-associated horse fecal microbiota. (**A**) Phylogenetic distribution of taxa with significant differences visualized via a branching graph. (**B**) Criteria for Scheme 3. (healthy: Group A; diarrheic: Group B).

At the genus level, *Lachnospiraceae*, *Christensenellaceae_R_7_group*, *Erysipelotrichaceae*, *NK4A214_group*, *Lachnospiraceae_XPB1014_group*, uncultured_rumen_bacterium, and *Candidatus_Saccharimonas* were the dominant bacteria in both groups (see Figure 3B). The distribution of and variability in fecal microbiota in diarrheic *P. przewalskii* horses are depicted by a heatmap (see Figure 4). The comparison between diarrheic and healthy Przewalski’s horses showed significantly increased abundances of *Christensenellaceae_R_7_group*, *Erysipelotrichaceae*, *Phoenicibacter*, and *WCHB1_41*, and significantly decreased abundances of *Lachnospiraceae_XPB1014_group*, *p_251_o5*, and *Lachnospiraceae_AC2044_group*. Combination of LEfSe and LDA scores was performed to further investigate the changes in fecal flora. In addition to the above differential taxa, diarrheic Przewalski’s horses also showed significantly higher abundances of Phoenicibacter and Coriobacteriales (Figure 5A,B).

### 3.4. Hormone and Immunoglobulin Assays

Analytical validation of the wild horse feces samples showed excellent accuracy. The results were plotted as the known standard dose versus apparent dose (the standard concentration minus added doses of the standard substance) with a slope of 1 (range 0.9–1.1), indicating that fecal components did not interfere with the accuracy of the determination at the detection dilutions. The within-batch coefficients of variation (CVs) of the control samples were as follows: Cor: 4.93%; T3: 7.32%; and immunoglobulin IgA: 7.63%. The between-batch CVs were as follows: Cor: 10.22%; T3: 14.74%; and immunoglobulin IgA: 13.68%. These values met the requirements of the method validation guidelines.

**Figure 6:**
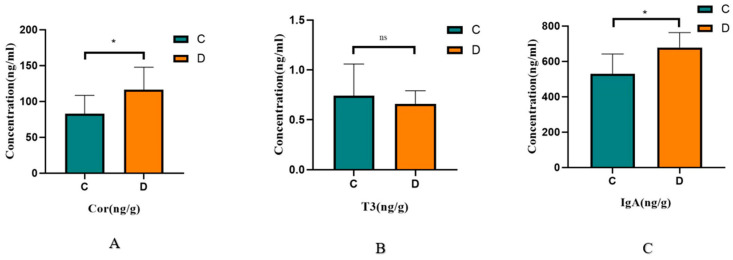
Comparison of hormone differences between diarrhea (Group D) and healthy (Group C) groups. (**A**) Cortisol; (**B**) triiodothyronine (T3); (**C**) immunoglobulin IgA (*, *p* ≤ 0.05, ns, *p* > 0.05).

Conducting hormone and immunoglobulin detection on the two groups of animals showed that the cortisol level in the diarrhea group was significantly higher than that in the normal group (*p* = 0.048, *n* = 7), there was no significant difference in the T3 level (*p* = 0.547, *n* = 7), and the IgA level in the diarrhea group was significantly higher than that in the normal group (*p* = 0.015, *n* = 7). The content of cortisol is an important indicator with which to measure the stress levels of animals. In this experiment, the cortisol level in the healthy group was significantly lower than that in the diarrhea group (*p* < 0.05). This indicates that diarrhea increases the stress level to a certain extent. T3 indicates the nutritional metabolism level of animals, and the experimental results did not reflect the impact of diarrhea on the nutritional metabolism level of animals; IgA indicates the intestinal immune level of animals, and the IgA level in the diarrhea group was increased, indicating that the body may be invaded by pathogens, thus increasing the IgA level (Figure 6A–C).

### 3.5. Correlation Network Analysis

**Figure 7:**
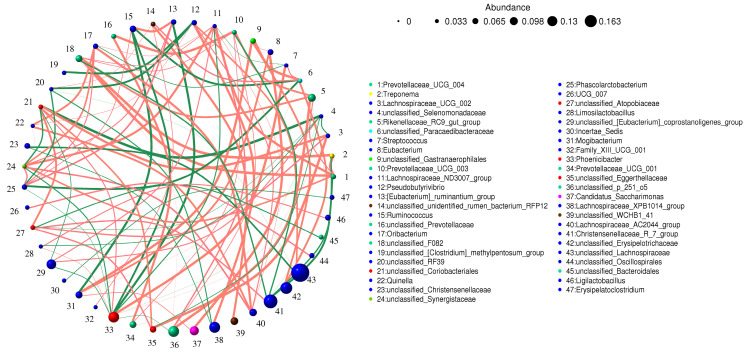
Network analysis shows connections between different bacteria. The red line indicates a positive correlation, and the green line indicates a negative correlation.

The results showed that Eubacterium was positively correlated with *Candidatus_Saccharimonas* (0.9164) and *Mogibacterium* (0.8769). *Lachnospiraceae_ND3007_group* was positively correlated with *Lachnospiraceae_AC2044_group* (0.8329) and *Oribacterium* (0.8329), *Prevotellaceae_UCG_004* (0.9076), [*Eubacterium*]_*coprostanoligenes_group* (0.8549), *p_251_o5* (0.8593), and *Synergistaceae* (0.8945). Limosilactococcus was positively correlated with *Ligilactobacillus* (0.8461) and *Phoenicibacter* (0.8329). *Phascolarctobacterium* was positively correlated with [*Eubacterium*]_*ruminantium_group* (0.8681) and *Ruminococcus* (0.8373). *Quinella* was positively correlated with *Lachnospiraceae_AC2044_group* (0.8769), *Oribacterium* (0.8769), *Synergistaceae* (0.8241), and *Pseudobutyrivibrio* (0.832) (Figure 7).

## 4. Discussion

Diarrhea is a disease that is particularly prevalent in wildlife, with a significant impact on the decline in wildlife populations [21,22]. The prevalence of diarrhea in Przewalski’s horses after their reintroduction to the wild in the Kalamaili Mountains Horse Reserve is high, posing a health risk to the reintroduction of Przewalski’s horses. However, several factors, including a harsh environment, nutritional imbalance, and the stress response, make the prevention and treatment of diarrhea in Przewalski’s horses particularly difficult. Recent studies on the fecal microbiota have highlighted its important role in the prevention and treatment of diarrhea [23,24]. The crucial role of gut microbiota in immunity, metabolism, and intestinal barrier function is widely acknowledged [25]. Hence, the elevated diarrhea rate among Przewalski’s horses may be attributed not only to their habitat environment but also to their intestinal flora. As members of the Equidae family, Przewalski’s horses are roughage herbivores adept at efficiently utilizing high-fiber grasses and other forages. The bulk of ingested plant fiber comprises structural carbohydrates like cellulose, hemicellulose, and lignin, which remain undigested by host enzymes in the foregut [26]. Undigested plant material reaches the hindgut where it undergoes microbial fermentation processes. These processes break down cellulose and hemicellulose, resulting in the production of energy-generating products such as short-chain fatty acids (SCFAs) [27]. It is estimated that a horse fed a forage-based diet can derive 50–70% of its energy requirements from short-chain fatty acids (SCFAs) [28]. In the arid environment of the Kalamaili Mountains, Przewalski’s horses require ample food to sustain their energy expenditure and support growth [29]. Our observations revealed a noteworthy reduction in bacteria, including those associated with cellulose degradation, during episodes of diarrhea, suggesting a diminished capacity for food digestion and degradation. Additionally, in the diarrhea group, IgA levels were elevated, indicating an activated immune response, while elevated cortisol levels pointed to increased stress and reduced immunity in diarrheic Przewalski’s horsess. Moreover, significant variations were observed among certain bacterial taxa in diarrheic Przewalski’s horses, underscoring their potential importance in maintaining intestinal microbial balance and intestinal function.

This study demonstrated that Firmicutes, Bacteroidetes, Actinobacteria, and Verrucomicrobia were the most dominant microbial phyla in Przewalski’s horses, regardless of health status. This finding aligns with previous studies on Przewalski’s horses [30]. Furthermore, these dominant phyla have been widely observed in other herbivorous animals such as goats, giraffes, and cattle, underscoring their importance in intestinal ecology and function across various species. Interestingly, our study revealed a significant reduction in certain bacterial genera in diarrheic Przewalski’s horses, including *Christensenellaceae_R_7_group*, *NK4A214_group*, *Lachnospiraceae_XPB1014_group*, *p_251_o5*, [*Eubacterium*]_*coprostanoligenes_group*, *Rikenellaceae_RC9_gut_group*, *Lachnospiraceae_AC2044_group*, *Ruminococcus*, and *Prevotellaceae_UcG_001*. These genera are recognized as beneficial bacteria in the intestine, known to play pivotal roles in enhancing digestion, metabolism, immunity, and fecal flora balance [31,32,33,34,35,36].

In fact, [*Eubacterium*]_*coprostanoligenes_group* is known to produce beneficial short-chain fatty acids (SCFAs) with anti-inflammatory effects. In addition, several of the bacterial genera mentioned above, including *Prevotella*, *Trichosporon*, and *Ruminococcaceae*, are recognized as SCFA producers. The SCFAs produced by these bacteria help to maintain gut health and exert anti-inflammatory properties, thus playing an essential role in gut homeostasis [25]. Previous studies have indicated a decrease in the abundance of SCFA-producing bacteria [37]. Indeed, SCFAs play multifaceted roles in regulating intestinal homeostasis, immunity, and barrier function. Additionally, they are crucial in mitigating inflammation and modulating energy intake [38]. SCFAs also play a vital role in inhibiting the proliferation of pathogenic bacteria, thereby enhancing the intestinal environment and contributing to disease prevention [39]. SCFAs play a multifaceted role in the regulation of several physiological processes. They facilitate communication between the gut and the brain through the brain–gut axis, modulating appetite and energy expenditure. In addition, SCFAs influence metabolic processes, contributing to wildlife adaptation to their environment. Additionally, by altering the pH of the gastrointestinal tract, SCFAs create an environment that is less favorable for the proliferation of pathogens, thereby aiding in disease prevention [40]. SCFAs have shown a strong correlation with cortisol and triiodothyronine T3. Specifically, SCFAs have been shown to stimulate the release of triiodothyronine T3, as well as serotonin (5-HT) and peptide YY (PYY). Peptide YY is a postprandial hormone involved in reducing appetite and intestinal motility [41,42]. Neuropeptides involved in appetite control and metabolism regulation may be influenced by the composition of the fecal microbiota. Studies have shown differences in microbiota composition between patients with diarrhea and healthy controls, suggesting a potential link between gut microbiota and the neuropeptide regulation of appetite and metabolism [43].

It is evident that there is a growing body of evidence demonstrating the intricate interplay between hormones and microbiota in immune responses, both in health and disease. There are numerous interconnections between them, and the microbiome and hormones can influence immune responses through shared pathways. This crosstalk between microbiota and hormones underscores the complexity of immune regulation and highlights the importance of considering these interactions in understanding immune function and disease pathology. High levels of intestinal microbial diversity and abundance are conducive to maintaining intestinal ecological balance and function. A diverse and abundant microbial community helps to prevent the overgrowth of potentially harmful bacteria, promotes the production of beneficial metabolites such as short-chain fatty acids (SCFAs), and enhances immune regulation. This equilibrium in the gut microbiota is of paramount importance for the overall health of the gut and plays a pivotal role in various physiological processes, including digestion, metabolism, and immune function [44]. It is indeed the case that a dysbiosis of intestinal microorganisms can impair the immune function of the intestine, thereby increasing the susceptibility to invasion by pathogenic and disease-causing bacteria. When the equilibrium of the gut microbiota is disrupted, it can result in an overgrowth of harmful bacteria and a decrease in beneficial bacteria, which in turn can lead to inflammation, compromised barrier function, and dysregulated immune responses. This dysbiosis creates an environment that is more conducive to the proliferation of pathogenic bacteria, thereby increasing the risk of infection and disease. It is therefore evident that maintaining a healthy balance of intestinal microorganisms is of paramount importance in order to support optimal immune function and overall health [45].

Diarrhea in Przewalski’s horses is associated with an increased risk of intestinal dysfunction and other diseases. This is thought to be due to a dysbiosis of the intestinal microbiota. β diversity analysis revealed significant differences in the major components of gut bacteria between healthy and diarrheic Przewalski’s horses, despite the fact that they share the same habitat and diet. This indicates that diarrhea may act as a fundamental driver of gut bacterial community change. Principal coordinate analysis (PCoA) was conducted to further elucidate the effects of diarrhea on the major components of gut microbiota in Przewalski’s horses. The results demonstrated that samples from healthy Przewalski’s horses formed a distinct cluster and were distinct from samples from diarrheic horses. Despite all selected Przewalski’s horses having identical diets and environments, alterations were observed in their fecal microbiota during episodes of diarrhea. Dysbiosis of the microbiota can significantly impact host immunity and gut reabsorption capacity, thereby increasing susceptibility to pathogens and predisposing individuals to various gastrointestinal diseases. This highlights the intricate relationship between the composition of the gut microbiota, the health of the host, and the susceptibility of Przewalski’s horses to gastrointestinal disorders [46,47].

Dysbiosis of the gut microbiome can contribute to the development of diseases characterized by hormone imbalances by modulating hormone levels. Glucocorticoids, such as corticosterone and cortisol, play a crucial role in modulating inflammation and have profound effects on both innate and adaptive immune responses. Dysbiosis-induced alterations in glucocorticoid levels can disrupt immune homeostasis, leading to dysregulated inflammatory responses and increased susceptibility to immune-related diseases. Consequently, maintaining a balanced gut microbiome is crucial for regulating hormone levels and supporting optimal immune function [48]. It is evident that, in addition to the suppression of pathogenic microorganisms, the fecal microbiota also plays a pivotal role in the regulation of immune responses, both locally within the gut and systemically throughout the body [48]. In the absence of commensal bacteria, the development of both the innate and adaptive immune systems of mice was impaired [49,50,51,52]. In this study, the levels of cortisol and immunoglobulin IgA in the diarrhea group were significantly higher than those in the healthy group. Cortisol, being a stress hormone, is known to suppress immune function, elevate physiological stress levels in animals, and consequently increase the susceptibility to intestinal infections [53,54]. Concomitantly, alterations in the intestinal bacterial community can result in increased intestinal mucosal permeability, thereby initiating an immune response. Consequently, this heightened immune response results in increased levels of serum IgA. Furthermore, the metabolism of the microbiome can influence the regulation of hormones involved in cholesterol, peptide, or amino acid production. Studies have demonstrated that elevated cortisol levels can impact the diversity of the intestinal microbiome in mice, potentially facilitating the invasion of intestinal pathogens such as Citrobacter rodentium into the body [55]. In a similar vein, our study revealed that cortisol levels were significantly elevated in individuals with diarrhea in comparison with those in healthy individuals. Conversely, intestinal beneficial bacteria were found to be significantly diminished in diarrheic Przewalski’s horses. It is postulated that this dysbiosis of the intestinal microbiota is a key driver of diarrhea in Przewalski’s horses. Furthermore, alterations in the relative abundance of specific bacteria within the intestinal tract may influence the functionality of other bacteria, thereby exacerbating alterations in the fecal flora.

Correlation network analysis revealed significant correlations among bacterial genera exhibiting significant changes, suggesting the potential for interactions that may weaken bacterial functions and affect overall intestinal function. This study demonstrates that diarrhea has a direct impact on the composition and diversity of fecal microorganisms, subsequently affecting the function of intestinal flora in Przewalski’s horses.

## 5. Conclusions

In conclusion, this study represents an inaugural investigation into the alterations in fecal microbiota and hormone levels in Przewalski’s horses with diarrhea. The results demonstrated that diarrhea was associated with elevated cortisol and IgA levels, accompanied by a significant reduction in fecal microbiota diversity and alterations in taxonomic composition. In particular, we observed a reduction in the proportion of beneficial bacteria and an increase in the abundance of pathogenic bacteria in fecal samples from horses with diarrhea.

This study addresses a significant gap in our understanding of the relationship between fecal microbiota and hormone levels in both healthy and diarrheal horses. It is of significant importance to note that this study highlights the potential role of intestinal microbial dysbiosis as a contributing factor to diarrhea in horses. Building upon these insights, our findings provide valuable implications for the prevention and treatment of diarrhea in Przewalski’s horses by targeting intestinal microbiota.

## Figures and Tables

**Table 1:** Bacterial sequence information for each sample.

Sample	Raw Reads	Clean Reads	Effective Reads	AvgLen	Q20 (%)	Q30 (%)	GC (%)	Effective (%)
CY1	80,135	79,935	78,718	410	52.9	99.35	97.1	98.23
CY2	80,097	79,898	78,957	412	53.06	99.33	96.99	98.58
CY3	79,993	79,796	78,385	411	53.03	99.34	97.04	97.99
CY4	79,870	79,681	78,252	410	52.85	99.36	97.11	97.97
CY5	79,962	79,767	78,507	412	52.69	99.36	97.09	98.18
CY6	80,151	79,966	79,105	411	52.89	99.37	97.16	98.69
CY7	80,106	79,913	79,090	411	53.35	99.35	97.08	98.73
DY1	80,007	79,808	78,941	411	53.24	99.33	97.04	98.67
DY2	79,988	79,805	78,733	411	53.46	99.34	97.07	98.43
DY3	80,256	80,054	78,555	411	53.53	99.36	97.16	97.88
DY4	79,970	79,764	77,554	411	53.59	99.32	96.97	96.98
DY5	80,174	80,002	78,609	411	53.24	99.34	97.05	98.05
DY6	80,051	79,833	79,364	406	53.66	99.34	97.08	99.14
DY7	80,017	79,807	78,545	411	53.32	99.35	97.07	98.16

## Data Availability

The data presented in this study are available on request from the corresponding author.
